# Editorial: The Role of lncRNA in Cancer Metabolism

**DOI:** 10.3389/fonc.2021.778068

**Published:** 2021-10-19

**Authors:** Guo-Liang Huang, Haiyan Hu, Xiao-Bin Lv

**Affiliations:** ^1^ China-America Cancer Research Institute, Guangdong Provincial Key Laboratory of Medical Molecular Diagnostics, Key Laboratory for Epigenetics of Dongguan City, Guangdong Medical University, Dongguan, China; ^2^ Oncology Department, Shanghai Jiao Tong University Affiliated Sixth People’s Hospital, Shanghai, China; ^3^ Jiangxi Key Laboratory of Cancer Metastasis and Precision Treatment, Central Laboratory, Nasopharyngeal Cancer Center, The First Hospital of Nanchang, The Third Affiliated Hospital of Nanchang University, Nanchang, China

**Keywords:** lncRNA, metabolism, glycolysis, oxidative phosphorylation, cancer

Metabolic reprogramming is a hallmark of cancer (1). Compared to normal cells, cancer cells undergo metabolic reprogramming to support their survival, proliferation, migration and metastasis. Alteration of enzymes such as HK2, PKM2, GLS1, IDH and SHMT2 is involved in cancer metabolic reprogramming. These enzymes are regulated by several oncogenic signaling pathways which play an important role in cancer metabolism (2). Recently, growing evidence indicates that long non-coding RNAs (lncRNAs) play an important role in many aspects of cancers including tumor cell metabolism (3). LncRNAs are mRNA-like transcripts that are longer than 200 nucleotides, yet do not appear to encode a protein. Many lncRNAs are differentially expressed between cancer tissues and corresponding para-cancer tissues, and their dysregulation has been connected to carcinogenesis and tumor progression in a variety of cancers (4). LncRNAs exert their function through miRNAs or directly through targeting metabolic reprogramming related kinases. Exploring the roles and mechanisms of lncRNAs in the regulation of cancer metabolism can contribute to a better understanding of cancer progression and to the discovery of novel therapeutic targets.

In this Research Topic, including two reviews and three original research articles, a comprehensive overview of the regulatory role of lncRNA in cancer metabolism was provided and novel function of several lncRNAs was revealed in cancer metabolism.

In their concise review, Ghafouri-Fard et al. summarized the most recent documents concerning the role of lncRNAs in cancer metabolism and related implication in cancer therapy. The function of lncRNAs could be divided into oncogenic or tumor suppressive in cancer. The role of oncogenic and tumor suppressor lncRNAs in cancer metabolism were listed by the authors. LncRNAs can exert their effects through different regulatory mechanisms such as modulating chromatin structure or DNA methylation status, interacting with transcription factors or DNA motifs, affecting mRNA processing, serving as miRNA sponges, and regulating alternative splicing processes. LncRNAs could be potential therapeutic targets of cancer.

Zhou et al. revealed the role of lncRNA-BLACAT1 in aerobic glycolysis and mitochondrial oxidative phosphorylation of pancreatic cancer cells. LncRNA-BLACAT1 was firstly identified as a novel long non-coding RNA in bladder cancer and was subsequently found to be a poor prognosis marker in several types of cancers. But few studies investigated the role of BLACAT1 in pancreatic cancer. In their study, Zhou et al. indicated that knockdown of BLACAT1 suppressed the cell proliferation, migration, aerobic glycolysis and tumor growth *in vivo*, and enhanced mitochondrial oxidative phosphorylation of pancreatic cancer. EZH2 is a H3K27 trimethylase that over-expressed in cancers. By recruiting EZH2, BLACAT1 promoted the H3K27 trimethylation of CDKN1C gene and modulated the expression of cyclin-E1 (CCNE) gene.

In their mini review, Kamada et al. focused the role of metabolism related lncRNA on breast and prostate cancers. Both breast and prostate cancers are two major types of sex hormone dependent cancers. both sex hormone-dependent and -independent pathways. Recent literatures regarding the role of metabolism related lncRNAs in the two cancers, focusing on both sex hormone-dependent and -independent pathways, were summarized in the review. In the regulation of glucose metabolism in breast or prostate cancers, several lncRNAs were found. HIF1α and MYC could cooperatively modulate the expression of glycolytic enzymes such as HK2, PFK, and LDHA. HIF1α was found to be regulated by lncRNA H19, LINK-A and lincRNA-p21. MYC was suggested to be regulated by lncRNA PCGEM1, FGF13-AS1. Concerning the lipid metabolism in breast or prostate cancers, lncRNA PCGEM1 could regulate the expression of several enzymes involved in lipid biosynthesis, such as FASN and ACACA.

The study by Tang et al. indicated that lncRNA DNAJC3-AS1 could regulate fatty acid synthase through the EGFR pathway and contribute to the development of colorectal cancer. DNAJC3-AS1 was firstly found to be over-expressed in colorectal cancer in multiple GEO datasets. Upregulation of DNAJC3-AS1 was confirmed in a cohort of independent cancer samples and was suggested to be a predictor of poor prognosis. DNAJC3-AS1 could enhance the proliferation, migration, invasion, and lipid accumulation of colorectal cancer cells. Expression of two fatty acid synthesis enzymes ACC1 and FASN was suggested to be modulated by DNAJC3-AS1 through EGFR/PI3K/AKT/NF-κB/SREBP1 signaling pathway.

Obesity is one of the risks for the development and progression in breast cancer. In order to identify potential biomarkers for adipocyte-associated-breast cancer, Cao et al. investigated the altered expression profile of lncRNA in breast cancer cells with the influence of adipocytes. Breast cancer cells MDA-MB-231 were co-cultured with human preadipocyte-visceral Hpa-V. Differential expression profiles of mRNA and lncRNA were identified between breast cancer cells with or without co-cultured with adipocytes. A total of 850 lncRNAs were found to be differentially expressed. After mRNA-lncRNA co-expression network was constructed, eight lncRNAs were suggested to be connected with the metabolism-related mRNAs. Moreover, the migration and invasion ability of breast cancer cells were enhanced with co-cultured adipocytes.

## Author Contributions

G-LH, HH, and X-BL are co-editors of this Research Topic. All authors equally wrote and contributed to the manuscript. All authors contributed to the article and approved the submitted version.

## Funding

G-LH was supported by National Natural Science Foundation of China (No: 81772982) and the Special Innovation Fund of Department of Education of Guangdong Province (No: 2019KTSCX049). X-BL was supported by grants from the National Natural Science Foundation of China (No. 81960501); the Natural Science Foundation of Jiangxi Province (No. 20202BAB206041); The foundation of Jiangxi Province Health Commission (20202003); and Grant from Nanchang science and Technology Bureau (2020-133).

## Conflict of Interest

The authors declare that the research was conducted in the absence of any commercial or financial relationships that could be construed as a potential conflict of interest.

## Publisher’s Note

All claims expressed in this article are solely those of the authors and do not necessarily represent those of their affiliated organizations, or those of the publisher, the editors and the reviewers. Any product that may be evaluated in this article, or claim that may be made by its manufacturer, is not guaranteed or endorsed by the publisher.
